# Machine Learning Approach to Identifying Wrong-Site Surgeries Using Centers for Medicare and Medicaid Services Dataset: Development and Validation Study

**DOI:** 10.2196/68436

**Published:** 2025-02-13

**Authors:** Yuan-Hsin Chen, Ching-Hsuan Lin, Chiao-Hsin Fan, An Jim Long, Jeremiah Scholl, Yen-Pin Kao, Usman Iqbal, Yu-Chuan Jack Li

**Affiliations:** 1 Department of Surgery Massachusetts General Hospital Boston, MA United States; 2 Center for the Evaluation of Value and Risk in Health Tufts Medical Center Boston, MA United States; 3 AESOP Technology Inc Taipei Taiwan; 4 Graduate Institute of Biomedical Informatics College of Medical Science and Technology Taipei Medical University New Taipei City Taiwan; 5 School of Population Health Faculty of Medicine and Health University of New South Wales Sydney Australia; 6 International Center for Health Information and Technology College of Medical Science and Technology Taipei Medical University Taipei Taiwan; 7 Department of Dermatology Wan Fang Hospital Taipei Taiwan; 8 Research Center for Artificial Intelligence in Medicine Taipei Medical University Taipei Taiwan

**Keywords:** patient safety, wrong site surgery, medical errors, machine learning, claim data

## Abstract

**Background:**

Wrong-site surgery (WSS) is a critical but preventable medical error, often resulting in severe patient harm and substantial financial costs. While protocols exist to reduce wrong-site surgery, underreporting and inconsistent documentation continue to contribute to its persistence. Machine learning (ML) models, which have shown success in detecting medication errors, may offer a solution by identifying unusual procedure-diagnosis combinations. This study investigated whether an ML approach can effectively adapt to detect surgical errors.

**Objective:**

This study aimed to evaluate the transferability and effectiveness of an ML-based model for detecting inconsistencies within surgical documentation, particularly focusing on laterality discrepancies.

**Methods:**

We used claims data from the Centers for Medicare and Medicaid Services Limited Data Set (CMS-LDS) from 2017 to 2020, focusing on surgical procedures with documented laterality. We developed an adapted Association Outlier Pattern (AOP) ML model to identify uncommon procedure-diagnosis combinations, specifically targeting discrepancies in laterality. The model was trained on data from 2017 to 2019 and tested on 2020 orthopedic procedures, using *ICD-10-PCS* (*International Classification of Diseases, Tenth Revision, Procedure Coding System*) codes to distinguish body part and laterality. Test cases were classified based on alignment between procedural and diagnostic laterality, with 2 key subgroups (right-left and left-right mismatches) identified for evaluation. Model performance was assessed by comparing precision-recall curves and accuracy against rule-based methods.

**Results:**

The findings here included 346,382 claims, of which 2170 claims demonstrated with significant laterality discrepancies between procedures and diagnoses. Among patients with left-side procedures and right-side diagnoses (603/1106), 54.5% were confirmed as errors after clinical review. For right-side procedures with left-side diagnoses (541/1064), 50.8% were classified as errors. The AOP model identified 697 and 655 potentially unusual combinations in the left-right and right-left subgroups, respectively, with over 80% of these cases confirmed as errors following clinical review. Most confirmed errors involved discrepancies in laterality for the same body part, while nonerror cases typically involved general diagnoses without specified laterality.

**Conclusions:**

This investigation showed that the AOP model effectively detects inconsistencies between surgical procedures and diagnoses using CMS-LDS data. The AOP model outperformed traditional rule-based methods, offering higher accuracy in identifying errors. Moreover, the model’s transferability from medication-disease associations to procedure-diagnosis verification highlights its broad applicability. By improving the precision of identifying laterality discrepancies, the AOP model can reduce surgical errors, particularly in orthopedic care. These findings suggest that the model enhances patient safety and has the potential to improve clinical decision-making and outcomes.

## Introduction

Wrong-site surgery (WSS) is recognized as the fifth most severe medical error in the United States [1]. A recent study revealed that one-third of inpatient WSS incidents resulted in high-severity injuries, with 7.4% of these cases leading to death [2]. This preventable mistake not only causes a big impact on patients but also imposes considerable financial burdens on the health care system [3]. According to a review in the United States National Practitioner Data Bank, a total of US $1.3 billion was recorded in payouts for surgical never events for 2 decades (1990-2010) [4]. Despite efforts aimed at reducing surgical errors through preprocedure verification, site marking, and a preoperative timeout, WSS events continue to occur. Specifically, the services most frequently responsible for those cases were orthopedic, including spine and intervertebral disc surgery, arthroscopy, and surgery on muscles or tendons [2,5].

The underreporting of WSS further complicates efforts to address this issue. In the United States, only 2% of sentinel events are reported to The Joint Commission, contributing to the persistence of WSS [1]. Moreover, the World Health Organization’s 2024 Global Patient Safety Report highlights that only 38% of countries have implemented systems to report preventable and catastrophic medical errors, leading to the widespread underestimation of their true frequency [6]. Among the factors to WSS, inconsistent documentation and inappropriate history and examination information contribute significantly to the occurrence of WSS [2,3]. For example, a patient may have a radiology report indicating a right renal mass from an outside hospital, while a referral document refers to a recent ultrasound report of a left renal mass, leading to a left nephrectomy [2]. Due to the inescapable nature of human error, the ability to automatically identify such discrepancies with the emerging technology could substantially reduce the incidence of WSS.

To address issues of inconsistent documentation and inappropriate history and examination information, a machine learning (ML) model that can automatically detect uncommon associations between diagnosis and surgical procedures may offer a viable solution for identifying and preventing WSS. Recent studies have shown promising results using ML algorithms to detect medication errors, using unsupervised association rule learning to identify unreasonable medication-disease combinations [7,8]. We hypothesized that the similar characteristics between procedure-diagnosis and medication-disease associations could be used to train a model capable of detecting surgical errors. Therefore, this study aims to assess the transferability of an adapted ML model and validate its performance in detecting inconsistencies in surgical procedure documentation.

## Methods

### Data Source

The Centers for Medicare and Medicaid Services Limited Data Set (CMS-LDS) files provide detailed information on health care services given to beneficiaries. This database includes claims data that record the medical services provided, along with related diagnosis and procedure codes [9]. This dataset is crucial for health care research and quality assessments, helping to measure health care outcomes in various settings [10-12]. We included claims with complete records of both medical and surgical procedures.

#### Model Construction

In this study, we constructed the adapted version of the Association Outlier Pattern (AOP) model using the ML algorithms, with methods described in a previous paper [13]. We used CMS-LDS data from 2017 to 2019 as a training set to develop the model. This AOP model is designed to automatically detect uncommon or rare associations between specific diseases and surgical procedures, with a particular focus on discrepancies between the diagnosed side (left or right) and the side operated on. The model determined a procedure to be substantiated if the index procedure could be explained by a relevant diagnosis. However, if there was a procedure that could not be explained by any of the diagnoses, then the procedure-diagnosis combination would be viewed as unsubstantiated. All data collection and analysis procedures in this study fully comply with the principles outlined in the “Guidelines for Developing and Reporting Machine Learning Predictive Models in Biomedical Research” [14].

#### Test Set Development

The study used inpatient CMS-LDS data from 2020 as a test set. We limited our testing data to orthopedic procedures, identified by the Attending Physician Specialty Code for Orthopedic Surgery. The study population was selected only if the *ICD-10-PCS* (*International Classification of Diseases, Tenth Revision, Procedure Coding System*) and *ICD-10-CM* (*International Classification of Diseases, Tenth Revision, Clinical Modification*) codes included identifiable body parts and laterality information. The fourth character of the *ICD-10-PCS* code was used to differentiate body parts and laterality. Cases in which the only procedure performed was the insertion of an infusion device were excluded, as the rationale for the insertion site is typically clinician-determined and often lacks a documented record.

Procedures and diagnoses were categorized by laterality: right, left, or both. “Right-side procedures” indicated that all procedures performed on the patient were on the right side, and “both-side procedures” indicated that the patient received both right- and left-side procedures. The same definitions applied to the laterality of diagnoses. Patients with complete laterality information for both procedures and diagnoses were classified into 9 subgroups based on the combination of procedural and diagnostic laterality, such as right-right, right-left, right-both, left-right, left-left, left-both, both-right, both-left, and both-both. The analysis focused on 2 subgroups with significant discrepancies between procedure and diagnosis laterality: right-side surgery with left-side diagnosis (right-left) and left-side surgery with right-side diagnosis (left-right).

#### Test Set Evaluation

The accuracy of these 2 subgroups was evaluated through a rigorous review process that examined the alignment between documented procedures and diagnoses. Two authors (YHC and CHL) independently reviewed the consistency between procedures and diagnoses. In cases of disagreement, both authors conducted a thorough secondary review of all available medical records and reached a consensus through discussion. A confirmed error case was defined as a scenario in which no direct association existed between the surgery performed and any recorded diagnosis. Clinical criteria for defining an error case included: (1) all diagnoses did not sufficiently justify the need for the surgery; (2) the anatomical areas mentioned in the diagnoses and procedures were either not the same, not in proximity (eg, shoulder joint and humerus are in proximity), or not appropriately related (eg, upper leg relates to femur bone); and (3) the anatomical areas were similar, but the laterality was opposite. These criteria ensured that discrepancies in laterality between diagnoses and surgical procedures were systematically identified.

Confirmed error cases were further categorized based on whether the procedure and diagnosis involved the same or different body parts. Confirmed nonerror cases were classified into five categories based on potential rationale: (1) same body part between procedure and diagnosis, but with the unspecified side in the diagnosis; (2) same body part between procedure and diagnosis, but with no laterality specified; (3) diagnosis without a specific body part that can explain the index procedure; (4) anatomical areas in the diagnoses and procedures were in proximity; and (5) the index procedure could be explained by a combination of diagnoses and concurrent procedures.

#### Model Performance Metrics

To assess the generalization and extrapolation performance of the model, we applied the trained AOP model to identify potentially unusual combinations of procedures and diagnoses among the 2 subgroups with significant discrepancies (right-left and left-right) in the test set. We plotted the precision-recall curves to obtain the optimal probability threshold to maximize the predictive capability of the AOP model. Precision-recall curves have been considered an effective metric for accessing the model, especially the data is an unbalanced dataset [15,16]. The optimal probability threshold is where a point can achieve high precision score while only sacrificing minimal recall (Figure S1 in Multimedia Appendix 1). By calculating the accurate prediction rate of the AOP model and the rule-based method (right-left and left-right), we can compare the performance between these 2 methods.

### Ethical Considerations

This study used the CMS-LDS files, which provide beneficiary-level information for research aimed at improving the US health care system. Access to the data was granted through the standard Centers for Medicare and Medicaid Services (CMS) approval process, including entering into a Data Use Agreement with CMS. The study was exempt from human subject research ethics review, as the data used were deidentified in accordance with the Health Insurance Portability and Accountability Act (HIPAA) Privacy Rule. Since the study involved secondary analyses of deidentified data, informed consent was not required, and no direct interaction with human participants occurred.

To ensure privacy and confidentiality, all analyses strictly adhered to CMS policies, including the cell suppression rule, which prohibits the publication of results with cell sizes of 10 or fewer (eg, patients or services). Furthermore, no attempts were made to reidentify individuals from the data, maintaining compliance with ethical and privacy standards.

## Results

### Baseline Study Population

**Figure 1:**
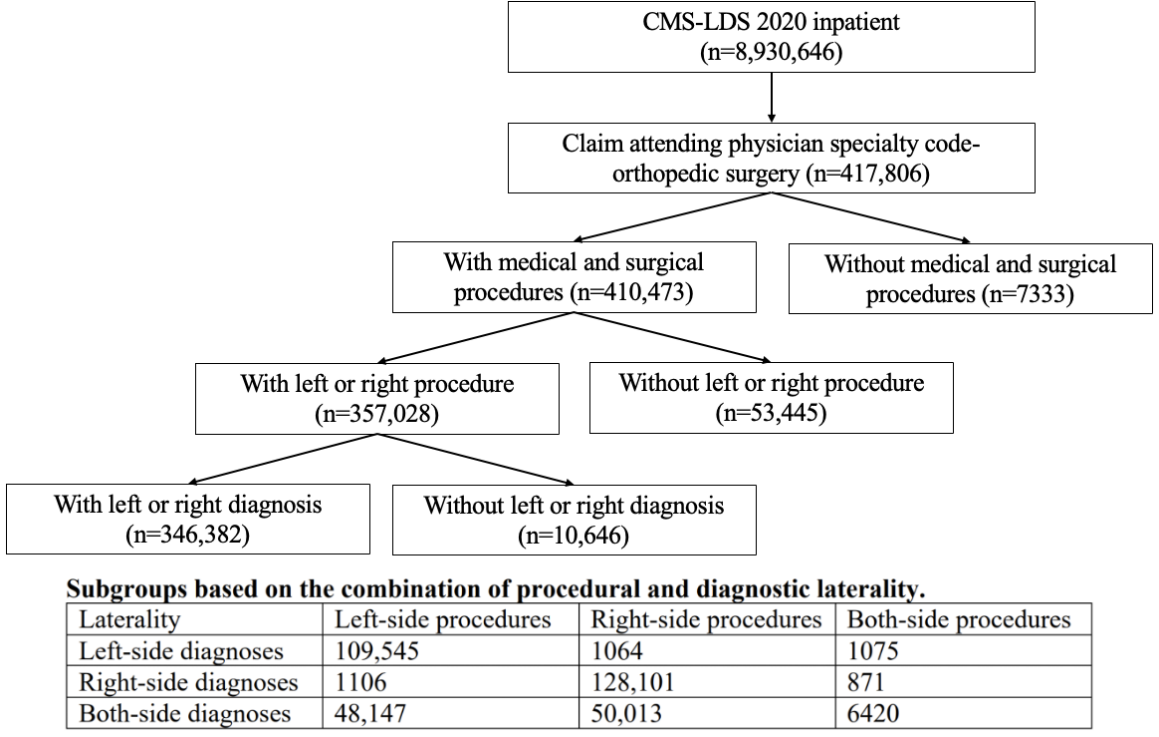
Selection of test set study population from Centers for Medicare and Medicaid Services Limited Data Set (CMS-LDS).

Data from a total of 346,382 claims were included in the study. Among them, the data indicated that 1106 patients had a left-side procedure with right-side diagnoses, and 1064 patients had a right-side procedure with left-side diagnoses (Figure 1).

The study used inpatient CMS-LDS data from 2020 as the test set. Testing data was restricted to orthopedic procedures, and identified using the Attending Physician Specialty Code for Orthopedic Surgery. The study population included only cases where the *ICD-10* (*International Statistical Classification of Diseases, Tenth Revision*) procedure and diagnosis codes provided clear information about the body part and laterality.

### Test Set Population Characteristics

Among the 1106 patients with left-side procedures with right-side diagnoses, 603/1106 (54.5%) cases were confirmed as errors, while 503/1106 (45.5%) were deemed reasonable combinations after applying the clinical review criteria. Similarly, among the 1064 patients with right-side procedures with left-side diagnoses, 541/1064 (50.8%) cases were confirmed as errors, while 523/1064 (49.2%) were deemed reasonable combinations (Table 1). Most confirmed errors involved the same body part in both procedure and diagnosis but with opposite laterality. Most nonerror cases were considered reasonable due to the presence of a relatively general diagnosis code with an unspecified body part, or a diagnosis with a specified body part but no laterality.

**Table 1:** Performance of rule-based method and association outlier pattern (AOP) model by laterality.

				Rule-based method^a^		AOP^b,c^ model
**Left-side procedure with right-side diagnoses**						
	**Confirmed error**			n=603 (54.5%)		n=576 (82.6%)
		Same body part	n=421		n=404	
		Not same body part	n=182		n=172	
	**Confirmed nonerror**			n=503 (45.5%)		n=121 (17.4%)
		Unspecified side of diagnosis^d^	n=39		n=17	
		No laterality for diagnosis^e^	n=131		n=34	
		No specific body part nor laterality^f^	n=251		n=58	
		Proximity area^g^	n=4		n=2	
		Others^h^	n=78		n=10	
**Right-side procedure with left-side diagnoses**						
	**Confirmed error**			n=541 (50.8%)		n=524 (80.0%)
		Same body part	n=390		n=376	
		Not same body part	n=151		n=148	
	**Confirmed nonerror**			n=523 (49.2%)		n=131 (20.0%)
		Unspecified side of diagnosis	n=50		n=17	
		No laterality for diagnosis	n=271		n=75	
		No specific body part nor laterality	n=129		n=25	
		Proximity area	n=4		n=4	
		Others	n=69		n=10	

^a^Left-side procedure with right-side diagnoses: n=1106; right-side procedure with left-side diagnoses: n=1064.

^b^Left-side procedure with right-side diagnoses: n=697; right-side procedure with left-side diagnoses: n=655.

^c^AOP: association outlier pattern.

^d^Same body part between procedure and diagnosis, but with the unspecified side in the diagnosis.

^e^Same body part between procedure and diagnosis, but with no laterality specified.

^f^Diagnosis without a specific body part that can explain the index procedure.

^g^Anatomical areas in the diagnoses and procedures were in proximity.

^h^The index procedure could be explained by a combination of diagnoses and concurrent procedures.

This table summarizes the performance of the rule-based method and the AOP machine learning model in identifying surgical procedure-diagnosis inconsistencies in the CMS-LDS from 2020. The study focused on patients undergoing orthopedic procedures with discrepancies in laterality between procedures and diagnoses. Results are presented for 2 subgroups: left-side procedures with right-side diagnoses (n=1106) and right-side procedures with left-side diagnoses (n=1064). Clinical review outcomes are categorized into confirmed errors and nonerrors, with further classification based on the nature of the diagnosis and its alignment with the index procedure.

We focused on 2 subgroups with significant discrepancies regarding laterality between procedure and diagnosis, where combinations could be reasonable or unreasonable. For instance, a patient who underwent right knee joint arthroplasty but was diagnosed only with left knee osteoarthritis was deemed to have an error, as no diagnoses could justify the need for right knee joint replacement. Conversely, a nonerror case might involve a patient who underwent right lower leg detachment surgery with a diagnosis of diabetes-related gangrene (Table 2). Although the diagnosis did not specify the body part or laterality, it could explain the reason for the surgery and did not conflict with other diagnoses.

**Table 2:** Test set evaluation examples for clinical review of procedure-diagnosis discrepancies.

		Index procedures	Diagnoses
**Confirmed error**			
	Same body part	Right knee joint 0SRC0J9-Replacement of right knee joint with synthetic substitute, cemented, open approach	M1712-Unilateral primary osteoarthritis, left knee F17210-Nicotine dependence, cigarettes, uncomplicated
	Not same body part	Left upper arm tendon, left shoulder joint 0LS40ZZ-Reposition left upper arm tendon, open approach 0RRK0JZ-Replacement of left shoulder joint with synthetic substitute, open approach	M19021-Primary osteoarthritis and right elbow E785-Hyperlipidemia, unspecified I10-Essential (primary) hypertension E7800-Pure hypercholesterolemia, unspecified
**Confirmed non-error**			
	Unspecified body part and unspecified laterality	Left hip joint 0SPB0JZ-Removal of synthetic substitute from left hip joint, open approach 0SRB049-Replacement of left hip joint with ceramic on polyethylene synthetic substitute, cemented, open approach	T8484XA-Pain due to internal orthopedic prosthetic devices, implants and grafts, initial encounter Y792-Prosthetic and other implants, materials and accessory orthopedic devices associated with adverse incidents S0501XA-Injury of conjunctiva and corneal abrasion without foreign body, right eye, initial encounter H10501-Unspecified blepharoconjunctivitis and right eye
	Unspecified body part and unspecified laterality	Right lower leg 0Y6H0Z2-Detachment at right lower leg, mid, open approach	E1152-Type 2 diabetes mellitus with diabetic peripheral angiopathy with gangrene I96-Gangrene, not elsewhere classified N186-End stage renal disease I69354-Hemiplegia and hemiparesis following cerebral infarction affecting left nondominant side E1122-Type 2 diabetes mellitus with diabetic chronic kidney disease E785-Hyperlipidemia, unspecified I252-Old myocardial infarction Z85528-Personal history of other malignant neoplasm of kidney
	Unspecified body part and unspecified laterality	Right femoral shaft and right upper femur 0QB80ZX-Excision of right femoral shaft, open approach, and diagnostic 0QH606Z-Insertion of intramedullary internal fixation device into right upper femur, open approach	C7951-Secondary malignant neoplasm of bone C3402-Malignant neoplasm of left main bronchus R200-Anesthesia of skin R591-Generalized enlarged lymph nodes
	No laterality for diagnosis	Left knee 0SPD08Z-Removal of spacer from left knee joint, open approach 0SRD0J9-Replacement of left knee joint with synthetic substitute, cemented, open approach	Z4733-Aftercare following explanation of knee joint prosthesis E785-Hyperlipidemia, unspecified I4510-Unspecified right bundle-branch block I10-Essential (primary) hypertension

This table provides representative examples of confirmed errors and nonerrors from the test set of orthopedic procedures in the CMS-LDS data from 2020. Confirmed errors include cases where procedural laterality (eg, left or right) did not align with the diagnosis, while nonerrors involve reasonable justifications for the procedure based on provided diagnoses. Each example lists the index procedures, associated diagnoses, and rationale for classification based on clinical review.

### AOP Model Performance Evaluation

Among the 1106 patients with left-side procedures with right-side diagnoses, the AOP model identified 697 potentially unusual combinations. After applying the clinical review criteria, 576 out of 697 (82.6%) cases were confirmed as errors, while 121 out of 697 (17.4%) were deemed reasonable. Similarly, among the 1064 patients with right-side procedures with left-side diagnoses, the AOP model identified 655 potentially unusual combinations. Of these, 524 out of 655 (80%) cases were confirmed as errors, and 131 out of 655 (20%) were reasonable combinations (Table 1). Most confirmed errors involved the same body part in both procedure and diagnosis but with opposite laterality. Most nonerror cases were considered reasonable due to a general diagnosis with an unspecified body part or a diagnosis with a specified body part but no laterality.

An example of a confirmed error among AOP model-identified cases was a patient who received right shoulder joint arthroplasty, while the diagnoses only included a left humerus fracture. Since a left arm fracture could not justify right shoulder joint replacement, this case was deemed an error. Conversely, an example of a nonerror case was a patient who underwent left glenoid cavity replacement, with a diagnosis of unspecified side rotator cuff tear or rupture (Table 3). As an unspecified side shoulder injury could justify left shoulder surgery, this case was deemed a nonerror.

**Table 3:** Association outlier pattern (AOP) model evaluation examples for identifying unusual procedure-diagnosis combinations.

			Index procedures		Diagnoses
**Confirmed error**					
	Same body part	Left hip 0SRB02A-Replacement of Left Hip Joint with Metal on Polyethylene Synthetic Substitute, Uncemented, Open Approach		M1611 - Unilateral primary osteoarthritis, right hip I10-Essential (primary) hypertension F329-Major depressive disorder, single episode, unspecified	
	Not same body part	Right shoulder joint 0RRJ0J6-Replacement of right shoulder joint with synthetic substitute, humeral surface, open approach		S42242A-4-part fracture of surgical neck of left humerus, initial encounter for closed fracture Z880-Allergy status to penicillin Z79899-Other long term (current) drug therapy	
**Confirmed non-error**					
	Unspecified body part and unspecified laterality	Right hip bursa 0MBL0ZZ-Excision of right hip bursa and ligament, open approach		T8131XA-Disruption of external operation (surgical) wound, not elsewhere classified, initial encounter G40909-Epilepsy, unspecified, not intractable, and without status epilepticus I110-Hypertensive heart disease with heart failure E669-Obesity, unspecified G2581-Restless legs syndrome Z96642-Presence of left artificial hip joint B964-Proteus (mirabilis) (morganii) as the cause of diseases classified elsewhere T8141XA-Infection following a procedure, superficial incisional surgical site, initial encounter I428-Other cardiomyopathies Y838-Other surgical procedures as the cause of abnormal reaction of the patient, or of later complication, without mention of misadventure at the time of the procedure	
	No laterality for diagnosis	Left glenoid cavity 0PR80JZ-Replacement of left glenoid cavity with synthetic substitute, open approach		M75120-Complete rotator cuff tear or rupture of unspecified shoulder, not specified as traumatic G8918-Other acute postprocedural pain M109-Gout and unspecified Z89511-Acquired absence of right leg below the knee	

This table illustrates specific examples of confirmed errors and nonerrors identified by the AOP machine learning model in the CMS-LDS 2020 test set. Confirmed errors are characterized by laterality discrepancies between the procedure and diagnosis, while nonerrors reflect plausible procedure-diagnosis relationships despite general or unspecified diagnoses. Each case includes the index procedures, associated diagnoses, and the rationale for classification.

## Discussion

### Principal Findings

In this study, we evaluated the effectiveness of the AOP model in detecting whether surgical procedures are substantiated with diagnoses records using CMS-LDS data. We found the AOP model is more accurate in identifying inconsistencies in surgical procedure documentation when compared with the traditional rule-based method. Meanwhile, the adapted AOP model also showed the transferability from detecting the medication-disease association and expanding to verifying the procedure-diagnosis association.

Our study adds to the existing literature that ML has penetrated the medical field with great success. ML allows for more detail to be mined from the data, allowing for the development of better diagnostic and prognostic tools than traditional approaches [17,18]. Similarly, the AOP model can detect associations between specific diseases and surgical procedures by learning patterns from a large database, while the rule-based method depends on the laterality of procedures and diagnoses. For example, among the 2 subgroups with significant discrepancies regarding laterality between procedure and diagnosis (left-right and right-left), a rule-based method classified all 2170 cases as inappropriate combinations due to the conflict in laterality. In contrast, the AOP model classified only 1352 of these cases as inappropriate. In terms of identifying inappropriate procedure-diagnosis combinations, the AOP model identified most errors similarly to the rule-based method (1100 for AOP vs 1144 for rule-based). In addition, the AOP performed with much higher accuracy (1100/1352, 81.4%) compared with the rule-based method (1144/2170, 52.7%).

The AOP model outperformed the traditional rule-based model, demonstrating a lower rate of false signals. Comparing the confirmed nonerror percentage between the rule-based method and the AOP model, there was a significantly lower rate of confirmed nonerror cases identified by the AOP model. In these cases, most confirmed nonerrors identified by the rule-based method could be explained by other diagnoses without mentioning body part or laterality. For example, cancer metastasis to bone may explain the reason for pelvic surgery, even without a specific pelvic metastasis diagnosis. This is because some diagnoses may not provide detailed information about the patient’s condition. Similarly, a patient who underwent lower leg detachment surgery with a diagnosis of diabetes-related gangrene could have the surgery justified, even if the gangrene diagnosis did not specify the body part. The rule-based method focuses solely on the laterality of the diagnosis, whereas the AOP model uses ML methods to explain the association between procedures and diagnoses. Therefore, when the association is deemed normal, the AOP model does not label them as inappropriate.

This study also showed several noteworthy findings. First, cases with fewer diagnoses were more likely to be labeled as inappropriate procedure-diagnosis combinations. For example, a patient who underwent surgeries for the left upper arm and left shoulder had only 4 diagnoses, including a right elbow problem and other chronic diseases. This may indicate incomplete diagnosis coding or an error in the surgical site. Second, the AOP model effectively identified unusual procedure-diagnosis combinations within subgroups where traditional rule-based methods failed to do so. Specifically, procedures that aligned with their corresponding diagnoses were deemed appropriate by conventional methods, such as left-side procedures with left-side diagnoses (left-left) and right-side procedures with right-side diagnoses (right-right). However, the AOP model continued to uncover atypical combinations within these groups. For instance, the model identified a patient who underwent left hip arthroplasty despite having a diagnosis of left knee osteoarthritis only.

The strengths of our study include its being the first to explore whether a ML model could play a surveillance role in detecting wrong site surgery. The effectiveness and fast pace to review the inconsistencies of surgical documentation help increase reporting mistakes in a timely manner without putting too much workforce on this issue. The findings from our study raise important questions regarding whether these errors stem from documentation mistakes or represent actual incidents involving patients. Therefore, in addition to retrospective reviews, it is essential to leverage modern technology to facilitate real-time error prevention. The potential of our model could add value on the current technology to encompass proactive prediction and analysis of diagnoses and clinical evidence to inform accurate surgical decisions and improve documentation practices [19]. In addition, the potential adaptability of the AOP model extends beyond orthopedic surgery to other specialties where procedural and diagnostic codes require logical alignment. For example, in ophthalmic surgery, the model could identify mismatches such as a diagnosis of left-sided cataract paired with a procedure for right-eye cataract surgery. Similarly, otolaryngology and other surgical fields with specific laterality could benefit. However, certain specialties may pose unique challenges due to differences in coding systems or the absence of laterality indicators, requiring further validation and specialty-specific refinements.

The AOP model has limitations. There were still nonerror cases identified by the AOP model. For instance, a patient who underwent left glenoid cavity replacement surgery with a diagnosis of unspecified rotator cuff tear was marked as inappropriate by the AOP model. However, the diagnosis could explain the necessity of the surgery. The reason the AOP model labeled this as an inappropriate combination may be due to the low frequency of this procedure-diagnosis combination in the CMS dataset. If the training dataset has a limited number of surgeries or diagnoses, then the association may be viewed as low and therefore marked as an inappropriate combination.

Another limitation is that the AOP model failed to identify some error cases labeled by the rule-based method. For example, a patient received an internal fixation surgery of the right upper femur with diagnoses of secondary malignant neoplasm of bone and left femur osteonecrosis. While the diagnosis of secondary malignant neoplasm could justify the surgical necessity, the diagnosis of left femur osteonecrosis conflicted with the right femur surgery. In such cases, the AOP model uses the maximum association within all procedure-diagnosis combinations, meaning that if the association between the surgery and a diagnosis is high, the model will view the combination as appropriate, even if the other diagnosis is in conflict. Therefore, in cases where one diagnosis can justify surgery, but another is conflicting, the AOP model may not accurately identify the combination. However, this aspect of the AOP model presents an opportunity for optimization, and future adjustments incorporating more detailed information through advanced ML techniques could yield a more accurate model, especially in cases where diagnoses are vague, incomplete, or lack clear specifications of body parts or sides.

Building on these strengths and limitations, future steps for implementing the AOP model in electronic health record systems could proceed along 2 directions. First, the model could support retrospective reviews by efficiently identifying WSS events, reducing review times and workforce burden, and enabling more timely reporting. Second, it could facilitate real-time consistency checks when clinicians input procedure codes. For example, if a surgeon enters a right-sided knee arthroplasty for a patient diagnosed with left knee osteoarthritis, the AOP model could flag the discrepancy and issue an alert. Such real-time notifications may prevent WSS before it occurs. However, to achieve successful implementation, the model must be optimized to address the limitations, and its performance must be validated across diverse electronic health record systems and datasets across different facilities.

### Conclusion

Overall, the AOP model enhances patient safety by systematically analyzing the cooccurrence frequency of diseases and surgical procedures on opposite sides, thereby identifying potentially unusual procedure-diagnosis combinations. Our analysis demonstrated a significant improvement in accuracy rates when the AOP model was used, compared with scenarios where it was not applied. By improving precision in identifying and validating cases with discrepancies between the diagnosed and operated sides, the AOP model has the potential to reduce the risk of errors in orthopedic treatment planning. This enhanced accuracy enables health care providers to make more informed decisions, reducing the risk of WSS and ultimately contributing to improved patient outcomes in orthopedic health care settings.

## Appendix

Multimedia Appendix 1 Supplement Figure 1. Precision-Recall curve of Association Outlier Pattern (AOP) Model in Test Set.

## Abbreviations

AOP: association Outlier Pattern
CMS: Centers for Medicare and Medicaid Services
CMS-LDS: Centers for Medicare and Medicaid Services Limited Data Set
HIPAA: Health Insurance Portability and Accountability Act
ICD-10: International Statistical Classification of Diseases, Tenth Revision
ICD-10-CM: International Classification of Diseases, Tenth Revision, Clinical Modification
ICD-10–PCS: International Classification of Diseases, Tenth Revision, Procedure Coding System
ML: machine learning
WSS: Wrong-site surgery

## Data Availability

This study used Centers for Medicare and Medicaid Services Limited Data Set (CMS-LDS) files. Access to these data is restricted and governed by CMS. Researchers interested in accessing the data must follow CMS’s application process and enter into a Data Use Agreement. Due to these restrictions, the data cannot be deposited in publicly available repositories. Requests for data access should be directed to CMS.
