# Supplementary figures and images for: Machine Learning Approach to Identifying Wrong-Site Surgeries Using Centers for Medicare and Medicaid Services Dataset: Development and Validation Study
Source: JMIR Form Res. 2025 Feb 13;9:e68436. doi: 10.2196/68436 (PMC11888080; doi:10.2196/68436)

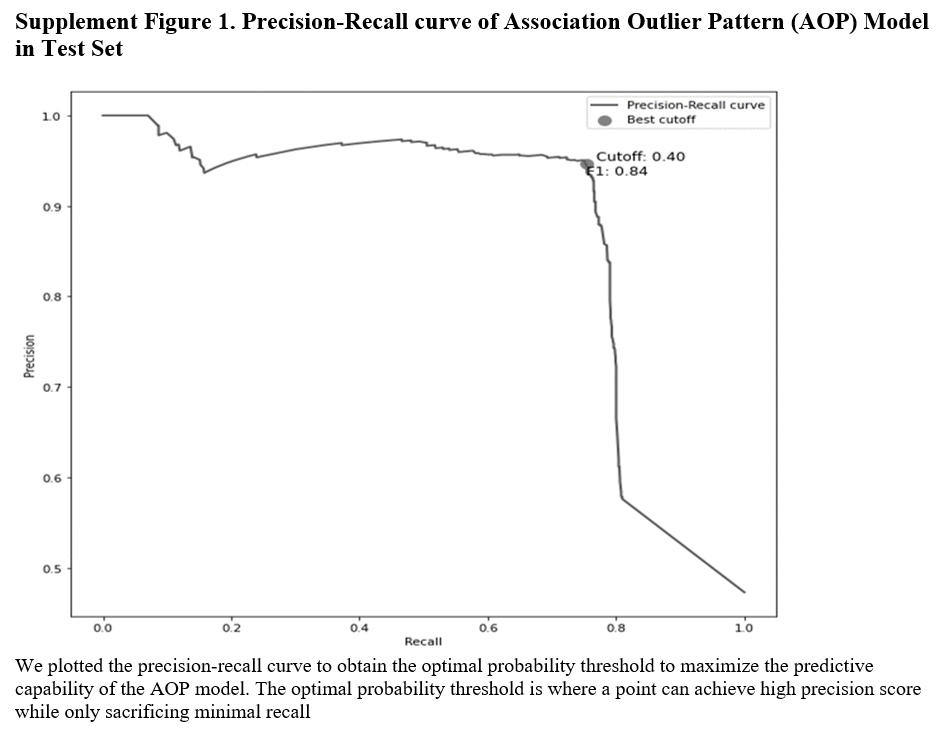

Supplement: Multimedia Appendix 1 [file formative_v9i1e68436_app1.png]
